# Signaling plasticity in the integrated stress response

**DOI:** 10.3389/fcell.2023.1271141

**Published:** 2023-12-07

**Authors:** Morgane Boone, Francesca Zappa

**Affiliations:** Bay Area Institute of Science, Altos Labs, Redwood City, CA, United States

**Keywords:** integrated stress response, signaling network, homeostasis, signal heterogeneity, subcellular compartmentalization, sensor kinase, signal crosstalk

## Abstract

The Integrated Stress Response (ISR) is an essential homeostatic signaling network that controls the cell’s biosynthetic capacity. Four ISR sensor kinases detect multiple stressors and relay this information to downstream effectors by phosphorylating a common node: the alpha subunit of the eukaryotic initiation factor eIF2. As a result, general protein synthesis is repressed while select transcripts are preferentially translated, thus remodeling the proteome and transcriptome. Mounting evidence supports a view of the ISR as a dynamic signaling network with multiple modulators and feedback regulatory features that vary across cell and tissue types. Here, we discuss updated views on ISR sensor kinase mechanisms, how the subcellular localization of ISR components impacts signaling, and highlight ISR signaling differences across cells and tissues. Finally, we consider crosstalk between the ISR and other signaling pathways as a determinant of cell health.

## Introduction

Stress signaling networks endow cells with the means to detect and react to homeostatic deviations. The Integrated Stress Response (ISR) is one such network; it adjusts protein biosynthetic rates and redirects resources to mitigate stress and restore homeostasis [reviewed in Pakos-Zebrucka et al. (2016); Costa-Mattioli and Walter (2020)]. However, if adaptive demands are unmet, the ISR switches to drive cell death, eliminating terminally injured cells for the benefit of the organism. The metazoan ISR has evolved a myriad of variations around this central operative tenet, which remains regulated by conserved building blocks found in all animals. In a nutshell, a battery of ISR sensor kinases transduces information about distinct stress states via phosphorylation of a common node, eIF2, a master regulator of protein synthesis, to terminal effectors that remodel the transcriptome and proteome.

In vertebrates, the ISR is governed by four sensor kinases that detect specific stress inputs: PKR (*EIF2AK4*), a sensor of double-stranded RNA (dsRNA) with a pivotal role in innate immunity; PERK (*EIF2AK3*), an ER-transmembrane protein that responds to ER homeostasis deviations; GCN2 (*EIF2AK2*) which detects amino acid availability and ribosome collisions; and HRI (*EIF2AK1*), which responds to heme depletion and recently has become recognized as a relay of mitochondrial stress (Donnelly et al., 2013; Pakos-Zebrucka et al., 2016; Costa-Mattioli and Walter, 2020). All these kinases converge on phosphorylating a single serine residue, Ser52 (conventionally referred to as Ser51), in the alpha subunit of the eukaryotic initiation factor 2 (eIF2α), a GTPase essential for translation initiation. The level of phosphorylated eIF2α regulates the availability of the ternary complex (TC), composed of eIF2, GTP, and the initiator methionyl-tRNA.

With initiation factors and the 40S ribosomal subunit, the TC forms the 43S pre-initiation complex (43S-PIC), which scans mRNAs in the 5′-to-3′ direction (Merrick and Pavitt, 2018). Recognition of the start codon by the 43S-PIC, combined with GTP hydrolysis by eIF2, triggers the release of GDP-bound eIF2 and a phosphate ion (Kapp and Lorsch, 2004; Algire et al., 2005; Majumdar and Maitra, 2005). The nucleotide exchange for GTP on eIF2 is catalyzed by eIF2B, eIF2’s dedicated guanine-nucleotide exchange factor (GEF), which enables TC recycling for every new round of translation initiation (Rowlands et al., 1988; Pavitt et al., 1998). Phosphorylated eIF2α acts as a potent inhibitor of eIF2B, thus directly regulating TC availability and global protein synthesis rates during stress encounters (Rowlands et al., 1988; Pavitt et al., 1998; Young and Wek, 2016; Adomavicius et al., 2019; Kashiwagi et al., 2019; Kenner et al., 2019; Schoof et al., 2021; Zyryanova et al., 2021).

As part of the ISR program, certain mRNAs are selectively translated in conditions where translation initiation rates are diminished due to low TC levels. These mRNAs are characterized by regulatory upstream open reading frames (uORFs) in their 5′UTRs and include those encoding the stress-regulated transcription factors ATF4 and CHOP (*DDIT3*), and GADD34 (*PPP1R15A*), a regulatory subunit of protein phosphatase 1 (PP1) (reviewed in (Young and Wek, 2016)). PP1-GADD34 dephosphorylates eIF2α, creating a negative feedback loop to terminate ISR signaling (Novoa et al., 2001). An additional non-stress-regulated PP1 regulatory subunit, CReP (*PPP1R15B*), constitutively dephosphorylates eIF2α to maintain steady-state protein synthesis (Jousse et al., 2003).

**FIGURE 1:**
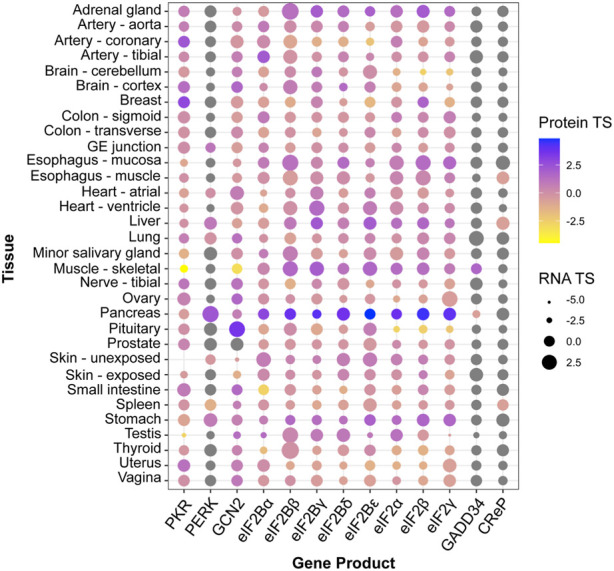
Tissue enrichment of core ISR components. Protein and RNA tissue specificity scores (TS) were extracted from the quantitative proteome map of the human body from the GTEx project, and are calculated as standard deviation from the bulk population average across all tissues for each gene product (Jiang et al., 2020). Grey dots: undetected by mass spectrometry. No data was available for ATF4 or HRI (EIF2AK1).

Despite its utility, the above-described unadorned view of the ISR remains reductionist and oversimplified. For instance, cellular differentiation implies that physiological ISR inputs, ISR components, and signaling pathways that crosstalk to the ISR, are likely to vary across cell and tissue types, leading to tailored outputs attuned to physiological demands. Indeed, genetic homozygous knock-out mouse models of core ISR components have divergent phenotypes. For example, *PKR* ^−/−^ mice develop normally, while knock-out of its sister kinase PERK leads to strong growth retardation (Table 1). Similarly, patients with ISR mutations can exhibit distinctive clinical manifestations [reviewed in English et al. (2022)]. Mutations that result in ISR activation, for instance, lead to growth impairment in the case of CReP, but leukodystrophy (known as Vanishing White Matter Disease) in the case of eIF2B. Conversely, disorders and conditions of vastly different etiologies present activation of the ISR (Costa-Mattioli and Walter, 2020). Furthermore, core ISR components can vary significantly in RNA and protein levels across tissues, which may lead to different ISR sensitivity (Figure 1; Anisimova et al., 2023). Accordingly, single-cell transcriptomes hint at vastly different ISR sensitivities of different brain cell types (Wong et al., 2019), and cell-type specific ISR signaling differences can be profound amongst cell types sharing ontogeny (Wolzak et al., 2022). Additionally, within the same cell type, emerging evidence suggests ISR regulation through spatial sequestration of components or stoichiometric regulation. We discuss these exciting findings in more detail below.

**TABLE 1:** Phenotypes of mice with homozygous deletions of key ISR components.

Official gene symbol	Protein	Constitutive null phenotype	References
*Eif2ak1*	HRI	viable and fertile; sensitive to iron deficient diet (anemia); increased susceptibility to *L. monocytogenes* infection; increased sensitivity to arsenite toxicity	Han et al. (2001), McEwen et al. (2005), Bahnan et al. (2018)
*Eif2ak2*	PKR	viable and fertile, same susceptibility to viral infection as WT; enhanced long-term memory; resistance to high-fat diet induced weight gain	Yang et al. (1995), Abraham et al. (1999), Nakamura et al. (2010), Zhu et al. (2011)
*Eif2ak3*	PERK	bone loss, diabetes, growth retardation	Harding et al. (2001), Zhang et al. (2002b), Wei et al. (2008)
*Eif2ak4*	GCN2	viable and fertile; increased mortality with aa-deprived diet; enhanced long-term memory	Zhang et al. (2002a), Anthony et al. (2004), Costa-Mattioli et al. (2005)
*Ppp1r15a*	GADD34	viable and fertile, hematopoietic defects, obesity (male mice), increased incidence of hepatocellular carcinomas, insulin resistance, NAFLD	Kojima et al. (2003), Nishio et al. (2014), Nishio and Isobe (2015), Nishio et al. (2017)
*Ppp1r15b*	CReP	growth retardation, impaired erythropoiesis	Harding et al. (2009)
*Eif2s1*	eIF2α	Embryonic lethal	https://www.mousephenotype.org/data/genes/MGI:95299
*Eif2s2*	eIF2β	Embryonic lethal	https://www.mousephenotype.org/data/genes/MGI:1914454
*Eif2s3x*	eIF2γ (X)	Viable and fertile^a^	https://www.mousephenotype.org/data/genes/MGI:1349431 ^a^
*Eif2s3y*	eIF2γ (Y)	Viable, infertile males	Mazeyrat et al. (2001), Matsubara et al. (2015)
*Eif2b3*	eIF2Bγ	Embryonic lethal	https://www.mousephenotype.org/data/genes/MGI:1313286
*Eif2b4*	eIF2Bδ	Embryonic lethal	https://www.mousephenotype.org/data/genes/MGI:95300
*Atf4*	ATF4	Low viability, growth retardation, bone loss, decreased insulin sensitivity, hematopoietic defects, eye defects	https://www.jax.org/strain/013072; Tanaka et al. (1998); Hettmann et al. (2000); Masuoka and Townes (2002); Lange et al. (2008); Yoshizawa et al. (2009)
*Ddit3*	CHOP	Viable and fertile, resistance to renal insufficiency and osteoarthritis after ER stress	https://www.mousephenotype.org/data/genes/MGI:109247; Zinszner et al. (1998); Yu et al. (2022)

^a^ Given its crucial role in the TC and its essentiality in human cell lines (https://depmap.org), eIF2γ is widely accepted as an essential gene in mammals. The source of the discrepancy with the viable phenotype in mice, reported by the International Mouse Phenotyping Consortium, remains to be investigated.

## Emerging views on sensor kinase signaling

### New sensing mechanisms

In the past decade, the repertoire of stress inputs detected by the ISR sensor kinases—directly, or indirectly through relay proteins—has expanded considerably. For HRI, it is well-established that its kinase domain can directly detect heme to coordinate the translation of globin mRNAs with heme and iron availability (Rafie-Kolpin et al., 2000; Han et al., 2001; Ricketts et al., 2022). However, it can function independently of heme to relay information about mitochondrial stress, ranging from redox imbalances to loss of mitochondrial proteostasis. This task is accomplished via interactions with the mitochondrial membrane protein DELE1, which is stabilized during mitochondrial stress and subsequently activates HRI in the cytosol (Fessler et al., 2020; 2022; Guo et al., 2020). DELE1 itself is also iron- (but not heme)- sensitive, providing another heme-independent path to HRI activation (Sekine et al., 2023). Moreover, HRI can be inhibited by the chaperone HSPB8, whose availability is regulated by specific pattern-recognition receptor “signalosomes” or amyloidogenic aggregates that serve as HSPB8 sinks (Abdel-Nour et al., 2019). These observations underscore the important role of HRI as a multi-stress information hub.

The reversible dissociation of the ER chaperone BiP (GRP78/HSPA5) from PERK during ER stress as a main mechanism controlling PERK activity has been re-evaluated based on biochemical and structural data, supporting a model wherein unfolded peptides serve as PERK-activating ligands (Wang et al., 2018), as occurs for the protein IRE1 (an ER stress sensor for the unfolded protein response) (Gardner and Walter, 2011; Karagöz et al., 2017). The emergent view highlights non-mutually exclusive activation models driven by protein-protein interactions. Other PERK activation mechanisms have surfaced, some of which appear independent of its classically defined lumenal ER stress sensor function. For one, ER-localized STING can transiently activate PERK on the cytosolic leaflet of the ER membrane, linking cGAS signaling and detection of cytosolic DNA to the ISR (Zhang et al., 2022). Similarly, ADP-ribosylation of PERK’s cytosolic domains by the tail-anchored protein PARP16 reportedly activates PERK without stress (Jwa and Chang, 2012). Acute cytosolic calcium depletion can activate PERK independently of its lumenal sensor domain; whether calcium levels directly modulate PERK remains to be determined (van Vliet et al., 2017). Finally, other non-proteinaceous PERK activators include changes in membrane lipid composition, which are thought to be detected through PERK’s transmembrane domain (Volmer et al., 2013).

PKR was initially identified as a sensor of viral dsRNA (Lemaire et al., 2008), but several studies indicate PKR also detects endogenous dsRNAs, expanding its roles beyond innate immunity. Indeed, PKR is activated by structured RNAs, including mitochondrial transcripts that leak upon mitochondrial damage, Alu and LINE1 transcripts, endogenous β-globin and TNF-α mRNAs, and small nuclear RNAs accessible during mitosis (Chu et al., 1998; Kim et al., 2014; Ilan et al., 2017; Namer et al., 2017; Kim et al., 2018). Moreover, a recent study suggests that PKR might sense misfolded IL-24 accumulating in the cytosol of cells with defective proteasomes (Davidson et al., 2022), broadening the repertoire of PKR inputs beyond dsRNA. Whether the IL-24/PKR interaction is direct and which PKR’s domain is implicated in this interaction is still unclear.

The GCN2 arm of the ISR is long-recognized to be engaged during amino acid deprivation (Berlanga et al., 1999; Sood et al., 2000), UV irradiation (Deng et al., 2002) and proteasomal inhibition (Jiang and Wek, 2005), all of which are likely to induce accumulation of uncharged tRNAs, which GCN2 directly recognizes (Wek et al., 1989; Dong et al., 2000; Padyana et al., 2005; Lageix et al., 2014). GCN2 also detects ribosome collisions through its His-tRNA synthetase-like and pseudokinase domains, even in the absence of deacetylated tRNAs, wherein specific ribosomal conformations expose the P-stalk of the ribosome (such as when ribosomes are stalled) to engage GCN2 (Ishimura et al., 2016; Harding et al., 2019; Inglis et al., 2019; Wu et al., 2020). Together, these observations support the notion of ISR kinase sensing polyfunctionality and a broad input repertoire for ISR kinases.

### ISR kinase promiscuity

The term “Integrated Stress Response” was coined two decades ago to describe the convergence of four stress sensor kinases acting on a common substrate, eIF2 (Harding et al., 2003). However, kinases are promiscuous, reflected by their relatively low number compared to all phosphoproteins in the cell (Johnson and Hunter, 2005). This raises the possibility that the ISR kinases phosphorylate more substrates than themselves—all ISR kinases autophosphorylate—and eIF2. Growing evidence supports this view. For example, PKR can phosphorylate the insulin-responsive protein IRS1 (Insulin Receptor Substrate 1), which negatively regulates insulin signaling, linking the ISR to glucose metabolism (Nakamura et al., 2010). GCN2 can phosphorylate and inhibit methionyl-tRNA synthetase (MARS1) in response to UV radiation, amplifying stress signals upon UV damage (Kwon et al., 2011). *In vitro* work suggests that human GCN2 can phosphorylate yeast eIF2β (Dokládal et al., 2021), although whether eIF2β is a *bona fide* target in mammalian cells remains confirmed. More recently, GCN2 was reported to phosphorylate PP1α and PP1γ, regulating their activity during mitosis (Stonyte et al., 2023). PERK purportedly phosphorylates the redox homeostasis transcription factor NRF2, leading to its dissociation from its inhibitor KEAP1 and enabling its nuclear translocation (Cullinan et al., 2003). Moreover, recent findings suggest that PERK could phosphorylate the autophagy proteins p62 and ULK1, leading to KEAP1 degradation and orthogonal control of NRF2 in hepatocytes exposed to lipotoxic stress (Lee et al., 2022). Interestingly, PERK has also been shown to regulate phosphatidic acid (PA) levels in an eIF2-dependent manner via the intramembrane PA transporter PRELID1, but also by directly phosphorylating diacylglycerol (DAG) to generate PA, thus impinging on the mTOR/Akt pathway (Bobrovnikova-Marjon et al., 2012; Perea et al., 2023).

### Sensor kinase signal modulation

Regulatory signaling modulation can also arise from interactions with ISR kinase regulators, which have been described for each ISR kinase. For example, the RNA binding protein TRBP inhibits PKR through physical interaction (Benkirane et al., 1997) and sequestration of the PKR activator PACT, which in turn controls PKR activity during recalcitrant ER stress (Singh et al., 2009). Similarly, the dsRNA deaminase ADAR1 also modulates PKR in a two-pronged manner: it directly inhibits PKR (Clerzius et al., 2009), but also suppresses endogenous dsRNA levels (Chung et al., 2018). In addition, early work shows PKR inhibition through direct interaction of its kinase domain with p58 (p58^IKT^/DNAJC3) (Lee et al., 1994; Gale et al., 1996; Polyak et al., 1996; Boriushkin et al., 2016), an interesting finding suggesting polyfunctionality and potential divergent subcellular localization—in the ER lumen and cytosol—of this BiP co-chaperone (Rutkowski et al., 2007; Tao et al., 2010), which can also inhibit PERK (van Huizen et al., 2003). PERK can also be negatively regulated by the Src-containing adaptor Nck1, which protects an inhibitory phosphoresidue in PERK’s cytosolic domain (Yamani et al., 2014). GCN2’s function is stimulated by GCN1, which directly detects ribosome collisions by binding to stalled disomes (Pochopien et al., 2021; Müller et al., 2023). Last, interactions between HRI and positive (DELE1) and negative (HSPA8) regulators can influence HRI signaling, as discussed above (Fessler et al., 2020; Guo et al., 2020).

### Newly identified ISR kinases

The complexity of a signaling network can be increased by expanding the repertoire of signal transducers. However, whether additional eIF2 kinases exist remains controversial. Quadruple knockout MEFs lacking PERK, HRI, PKR, and GCN2 (4KO) do not show any signs of eIF2α phosphorylation under acute stresses (Taniuchi et al., 2016), suggesting that the ISR exclusively hinges on its four recognized stress sensor kinases in the initial phase of the response. However, recent evidence suggests that the kinase MARK2, a microtubule-associated kinases (MAPKs) regulator, directly phosphorylates eIF2α in response to chronic proteotoxic stress, even in 4KO cells (Lu et al., 2021). These findings point to the activation of compensatory mechanisms to ensure ISR signaling upon enduring stress. MARK2-mediated phosphorylation of eIF2 requires HSP90 and the isoform δ of protein kinase C (PKCδ), which activate MARK2, making MARK2 a stress information relay. Moreover, the brain-specific kinase FAM69C (*DIPK1C*) has been shown to directly phosphorylate eIF2α *in vitro*, and its loss-of-function results in ISR defects in microglia exposed to oxidative stress (Wu et al., 2023). However, the annotated localization of FAM69C’s kinase domain in the ER lumen suggests that the *in vivo* regulation of eIF2α by FAM69C might be indirect.

## Spatial organization of ISR components within cells

The spatial organization and association of molecular components within cells create unique microenvironments that serve as crucibles for biochemical reactions. Likewise, ISR nodes populate specific cellular locales, and ISR kinase activity is regulated by self-association. Several lines of evidence support the notion that active PERK forms high-order oligomers in the plane of the ER membrane (Bertolotti et al., 2000; Carrara et al., 2015; Sundaram et al., 2018; Wang et al., 2018), but the distribution of inactive PERK and PERK oligomers is poorly understood. PERK is also found at ER-mitochondrial contact sites (EMCS) (Verfaillie et al., 2012; Muñoz et al., 2013; van Vliet et al., 2017), and new details about its function and regulation at this specific location have recently been revealed. PERK can act as an adaptor to recruit the lipid-transfer protein E-Syt1 (extended synaptotagmin 1) to EMCS to control mitochondrial respiration; whether this interaction modulates the ISR remains unexplored (Sassano et al., 2023). During ER stress, a covalent interaction between PERK and ERO1α increases the number of EMCS and is essential to improve calcium flux between both organelles (Bassot et al., 2022). Moreover, an inhibitory interaction between PERK and the mitochondrial membrane protein ATAD3 at EMCS allows bypassing ISR translational regulation of transcripts encoding mitochondrial proteins even in the absence of deacetylated tRNAs (Hughes et al., 2022).

The ER provides a platform for the spatial organization and regulation of other ISR components beyond PERK. The constitutive eIF2α phosphatase CReP (PPP1R15B) also associates with the ER-membrane (Kloft et al., 2012), enabling selective protein synthesis of membrane-tethered transcripts despite ISR induction (Kastan et al., 2020). GADD34 (PPP1R15A), the stress-induced counterpart of CReP, can be inserted in the cytoplasmic leaflet of the ER membrane, a process that controls its availability by proteasomal degradation (Brush et al., 2003; Zhou et al., 2011).

In the cytosol, PKR partitions to membrane-less compartments upon detection of dsRNA. These PKR coalescences exclude eIF2α, indicating that they temper enzyme-substrate interactions. Indeed, disruption of PKR clustering enhances ISR signaling, suggesting that PKR utilizes subcellular compartmentalization to limit interactions with eIF2 and buffer ISR signaling (Corbet et al., 2022; Zappa et al., 2022). HRI self-associates into higher-order oligomer assemblies upon activation *in vitro* (Bauer et al., 2001; Miksanova et al., 2006; Bhavnani et al., 2018). Whether HRI forms coalescences in cells remains to be investigated. Recent structural findings have revealed that DELE1 organizes into an octamer capable of modulating HRI activity, raising the possibility that DELE1 multimers may template HRI multimerization to facilitate autophosphorylation (Yang et al., 2023).

Phosphorylation of eIF2α and the ensuing suppression of translation initiation stimulates the formation of stress granules (SGs); cytosolic, phase-separated, membrane-less compartments containing mRNA and various components of the translational machinery. SGs serve as reservoirs, protecting transcripts from degradation during stress, and act as signaling hubs (Banani et al., 2017; Youn et al., 2019). For instance, the master ISR transcription factor ATF4 is selectively translated in SGs (Mateju et al., 2020), and components of the ER-export machinery are sequestered in SGs to limit secretion during ISR induction, further supporting the roles of SGs as ISR regulators (Zappa et al., 2019). Moreover, in neurons of Alzheimer’s disease brains, both ATF4 protein and mRNA are enriched in axons when compared to the soma, raising the appealing possibility that local synthesis might direct interaction with specific binding partners (Baleriola et al., 2014).

Another example of subcellular compartmentalization in the ISR involves eIF2B. In yeast, eIF2B forms filamentous structures where eIF2 exchanges rapidly within the cytosolic pool (Campbell et al., 2005; Marini et al., 2020; Nüske et al., 2020), and in mammalian cells, eIF2B subunits are found in cytoplasmic foci of varying sizes, even in the absence of stress (Hodgson et al., 2019; Hanson et al., 2023). The dynamic shuttling of eIF2 into eIF2B-containing structures reflects eIF2B activity, suggesting that these foci are enzymatic factories for eIF2 activation. These findings indicate that the reversible formation of condensates and the subcellular localization of ISR components may enable precise control and coordination of the ISR.

## Convergence and connectivity: interplay between the ISR and other signaling networks

### Crosstalk to other signaling pathways

Stress inputs simultaneously activate multiple intertwined pathways that make up complex signaling networks. The ISR is not exempt from this paradigm, and recent evidence supports the notion that it is deeply interconnected with many signaling pathways. Indeed, the many ways all four arms of the ISR communicate with innate and adaptive immune signaling pathways have been extensively reviewed elsewhere (Martins et al., 2016; Reverendo et al., 2019; Salvagno and Cubillos-Ruiz, 2019; Costa-Mattioli and Walter, 2020; Girardin et al., 2021; Zhao et al., 2023). Specific examples include the modulation of PKR by interferons (Meurs et al., 1990) and the stimulation of NF-kB signaling by PKR (Bonnet et al., 2000; Zamanian-Daryoush et al., 2000); the critical roles of HRI in tuning NOD1 and NOD2—bacterial peptidoglycans sensors that activate NF-κB (Abdel-Nour et al., 2019); and the feed-forward loop resulting from IL-6 induction by ATF4 (Iwasaki et al., 2014).

Beyond immunity, crosstalk can occur through modulatory interactions contingent on subcellular localization. In the ER, unfolded proteins activate PERK, as well as IRE1, and ATF6, two key ER stress sensors. PERK induces the phosphatase RPAP2, which dephosphorylates IRE1 (Chang et al., 2018), establishing negative feedback control to suppress IRE1 pro-survival signals and favor PERK-dependent apoptosis (Chang et al., 2018; Muniozguren et al., 2022). Similarly, ER stress induces BiP downstream of ATF6 and IRE1, thereby exerting negative feedback control of PERK—and IRE1 and ATF6—activities to resolve ER stress (Wang et al., 2000). Interconnectivity also stems from the linkage of ER-associated and cytosolic processes. For example, extrusion of unfolded IL-24 from the lumen of the ER into the cytosol activates PKR (Davidson et al., 2022); and an overwhelmed ubiquitin-proteasome system, as observed in Parkinson’s disease, can activate PERK (Zhang et al., 2010; Hughes and Mallucci, 2019).

The ISR is intimately connected with the cell’s master regulator of anabolic and catabolic processes: the mTOR (mechanistic Target Of Rapamycin) signaling pathway. Amino acid deprivation activates the ISR while suppressing mTORC1 signaling, resulting in exquisite control of protein biosynthetic rates (Liu and Sabatini, 2020). Interestingly, mTORC1 can regulate ATF4 levels in a phospho-eIF2-independent manner by enhancing ATF4 mRNA stability and basal translation through increased mRNA cap-binding protein eIF4E availability (Park et al., 2017; Torrence et al., 2021).

The ISR and mTOR signaling networks are further intertwined through the regulation of autophagy. Deactivation of mTORC1 licenses lysosomal biogenesis and enhances lysosomal function by activating the TFEB family of transcription factors (Settembre et al., 2012). TFEB induces ATF4 and GADD34 mRNA expression, thereby engaging ATF4-driven biosynthetic programs coupled to the negative feedback loop that shuts down ISR signaling (Martina et al., 2016). In this way, during amino acid starvation, the cell ensures the translation of mRNAs encoding autophagy regulators induced by TFEB that require escaping the negative translational control imposed by the ISR (Gambardella et al., 2020). Notably, the ISR gene expression program encompasses autophagy genes, and indeed, eIF2α phosphorylation is required to induce autophagy upon treatment of cells with many pharmacological agents (Humeau et al., 2020). In addition, PERK induces the autophagy regulator ATG12 and stimulates the expression and activation of the autophagosome formation mediator of LC3 (Kouroku et al., 2007); PKR engages autophagy through activation of IKKβ and JNK1, and phosphorylation of IRS1, a known autophagy inducer (Nakamura et al., 2010); and HRI induces BAG3 and HSPB8, key components of chaperone-assisted selective autophagy of cytosolic proteins (Mukherjee et al., 2021). As such, the ISR coordinates catabolism through inhibition of mRNA translation, and by regulation of amino acid recycling through autophagy.

Additional translation modulators significantly influence transcriptional reprogramming during the ISR, and hence may contribute to signaling plasticity across tissues and conditions. The translation factors EIF2D, MCTS1, and DENR were shown to be required for ATF4 translation through their effects on ribosome recycling in both *Drosophila* (flies) and human cells (Bohlen et al., 2020; Vasudevan et al., 2020; Young et al., 2021). In addition, translation of the ATF4 mRNA is also regulated by RNA methylation (Zhou et al., 2018), an event that is modulated by eIF3D during chronic stress (Mukhopadhyay et al., 2023). These studies underscore the importance of precise translational control during ISR.

### Cell-type-specific ISR wiring

Cell identity and the ensuing intrinsic physiological demands require tailored ISR responses, some of which have come to light recently. For example, orthogonal studies in unstressed, wild-type mice have revealed intrinsic ISR signatures in forebrain astrocytes and Bergmann glia in the cerebellum (Wong et al., 2019), and in cholinergic interneurons in the striatum (Helseth et al., 2021), suggesting that these cells rely on the ISR for basic functions. Further evidence suggests distinct ISR outputs in neurons and astrocytes, wherein lack of PERK is compensated by HRI in astrocytes but not in neurons (Wolzak et al., 2022). Moreover, neurons and astrocytes exhibit differences in the localization and composition of eIF2B complexes, and their ISR outcomes vary when subjected to chronic stress and ISR inhibition (Hanson et al., 2023). Inactivating mutations in eIF2B, which are associated with Vanishing White Matter Disease, affect astrocytes and oligodendrocytes prior to neuron dysfunction (Dietrich et al., 2005; Dooves et al., 2016; Wisse et al., 2018; Zhou et al., 2019; Deng et al., 2022). Along this line, ISR modulation can have dramatically different results in neuronal cell types: adjusting the ISR in excitatory and somatostatin-positive inhibitory neurons, but not parvalbumin-positive inhibitory neurons, results in altered long-term memory consolidation (Sharma et al., 2020; Shrestha et al., 2020). Another example highlights the differences in the activation threshold of the ISR in different neural cell types: the GCN2 inhibitor IMPACT is significantly more abundant in hypothalamic neurons than in other cells (Pereira et al., 2005; Bittencourt et al., 2008), which could lead to distinct regulation of the ISR in conditions that activate GCN2.

Beyond the brain, in the mouse and human intestinal epithelium, secretory goblet cells can readily adapt to prolonged ER stress, but absorptive enterocytes are prone to ISR-induced expression of the transcription factor QRICH1, which promotes sustained, unmanageable protein synthesis that leads to cell death (You et al., 2021). Myoblasts and myotubes differ in their ability to mount an ISR during mitochondrial electron transport chain dysfunction due to differences in their metabolic state (Mick et al., 2020). In the testis, increased protein synthesis during spermatogenesis requires an alternative eIF2γ subunit (*Eif2s3y* in mice, *EIF2S3B* in humans) (Mazeyrat et al., 2001; Matsubara et al., 2015). All these examples underscore the notion that circuit complexity within the ISR enables cell type-specific adaptations.

## Discussion

Specific cellular physiological demands call for distinct ISR adaptations in multicellular organisms. Classic examples include the endocrine pancreas’ reliance on PERK, erythroblasts’ dependence on HRI’s heme-sensing, and the dysregulation of ISR in neuropathology (Kefalas and Larose, 2018; Chen and Zhang, 2019; Bond et al., 2020). Beyond these examples, an emerging constellation of small molecules, lipids, and protein activators of ISR kinases, alternate ISR kinases, additional substrates, and ISR core component availability mediated by expression, stability, enzymatic activity, and spatial sequestration, portrays a complex and intricate network of mechanisms through which cells modulate the ISR with finesse. Speculations abound around this theme, leading us to postulate here less-explored hierarchies of ISR modulation, ranging from metabolite, lipid, and post-translational-modification (PTM)-mediated ISR regulation, to ISR tuning by intercellular communication and ISR molecular memories.

We surmise that many metabolites could affect the ISR, as occurs with fructose-6-phosphate, an enhancer of eIF2B activity (Hao et al., 2021). Moreover, defects in lipid metabolism caused by expression of the 4ε isoform of the lipid transporter APOE activate the ISR (Segev et al., 2013; 2015), suggesting lipid-mediated mechanisms of ISR regulation. Many core components of the ISR are extensively post-translationally modified, implying that PTMs may exert exquisite control to diversify and adjust ISR outputs. For instance, ATF4 is extensively regulated by phosphorylation, methylation, and ubiquitination, which can impact its protein stability and activity (Yang et al., 2004; Frank et al., 2010; Kim et al., 2011; Fan et al., 2014; Yuniati et al., 2016). Similarly, eIF2 and eIF2B can be phosphorylated and acetylated on multiple subunits (Welsh et al., 1998; Wang et al., 2001; Beilsten-Edmands et al., 2015). However, the exact function, regulation, and integration of these PTMs remain poorly understood.

Cell-to-cell communication could endow the ISR with an additional layer of regulation with the potential to impact cellular decisions within cell “ecosystems” and, consequently, the health of tissues and organs. Accumulating evidence suggests adaptive and terminal stress responses propagate signals amongst cell communities to coordinate cell responses across tissues. For example, apoptotic cells secrete proteins and metabolites that allow the transmission of information across spatial scales (Christiansen et al., 2013; Pérez-Garijo et al., 2013; Medina et al., 2020), cells experiencing mitochondrial stress inform neighboring cells via mitokines (Durieux et al., 2011; Berendzen et al., 2016; Shao et al., 2016; Zhang et al., 2018; Lan et al., 2019; Kang et al., 2021), and ER stress signaling in the nervous system induces ER stress responses in peripheral tissues in nematodes and vertebrates (Taylor and Dillin, 2013; Williams et al., 2014; Imanikia et al., 2019; Daniele et al., 2020). These observations suggest that the ISR may engage similar cell non-autonomous mechanisms, especially considering that the ISR induces the expression of chemokines and can trigger apoptosis (Hattori et al., 2003; Iwasaki et al., 2014; Forsström et al., 2019; Sprenkle et al., 2019).

Another aspect that requires further investigation is the mechanistic characterization of an ISR molecular memory that could stand behind cell resilience and the hormetic adaptation of tissues and organs. Hints of such adaptive stress memories have been observed in *C. elegans,* in which exposure to oxidative stress early in life protects older worms from heat shock (Bazopoulou et al., 2019; Zhang et al., 2021). Closer to the ISR, GADD34 has been suggested to play a central role in cellular adaptation to repetitive stresses (Batjargal et al., 2022; Klein et al., 2022). Whether epigenetic ISR memories exist and how they contribute to heritable hormetic adaptation remains to be defined.

The broad capacity of the ISR to multitask and control cellular decisions supports a model in which the ISR could be envisioned as a central integrator of signaling pathways controlling the homeostatic capacity of the cell. Such a network would allow finely tuned responses tailored to meet the specific demands imposed by the intrinsic physiology of cells and tissues. While much progress has been made to unearth new roles and nuances of the ISR signaling network, it has also made it clear that the ISR will continue to yield surprises for decades to come.
